# Silicon-Based Solid-State Batteries: Electrochemistry and Mechanics to Guide Design and Operation

**DOI:** 10.1021/acsami.3c06615

**Published:** 2023-08-30

**Authors:** Pooja Vadhva, Adam M. Boyce, Anisha Patel, Paul R. Shearing, Gregory Offer, Alexander J. E. Rettie

**Affiliations:** † Electrochemical Innovation Lab, Department of Chemical Engineering, University College London, London WC1E 7JE, United Kingdom; ‡ School of Mechanical and Materials Engineering, University College Dublin, Dublin, D04 V1W8, Ireland; § Department of Mechanical Engineering, Imperial College London, London SW7 1AY, United Kingdom; ∥ The Faraday Institution, Quad One Becquerel Avenue Harwell, Didcot OX11 0RA, United Kingdom

**Keywords:** solid-state battery, thin film, solid electrolyte, material selection, finite element analysis model, elastic, plastic, silicon negative electrode

## Abstract

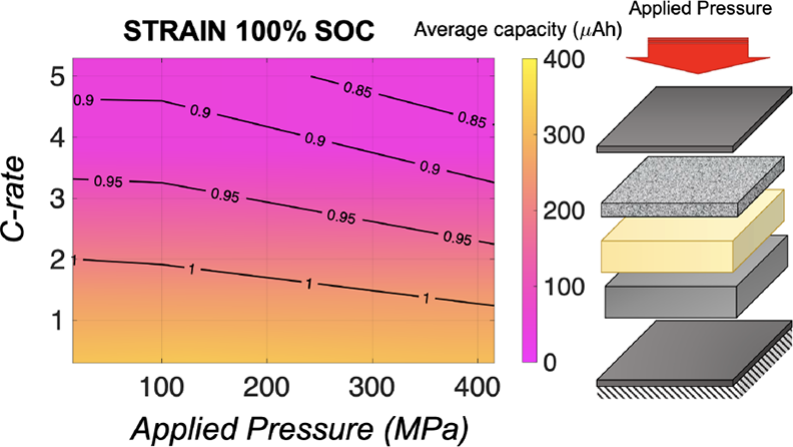

Solid-state batteries (SSBs) are promising alternatives to the incumbent lithium-ion technology; however, they face a unique set of challenges that must be overcome to enable their widespread adoption. These challenges include solid–solid interfaces that are highly resistive, with slow kinetics, and a tendency to form interfacial voids causing diminished cycle life due to fracture and delamination. This modeling study probes the evolution of stresses at the solid electrolyte (SE) solid–solid interfaces, by linking the chemical and mechanical material properties to their electrochemical response, which can be used as a guide to optimize the design and manufacture of silicon (Si) based SSBs. A thin-film solid-state battery consisting of an amorphous Si negative electrode (NE) is studied, which exerts compressive stress on the SE, caused by the lithiation-induced expansion of the Si. By using a 2D chemo–mechanical model, continuum scale simulations are used to probe the effect of applied pressure and C-rate on the stress–strain response of the cell and their impacts on the overall cell capacity. A complex concentration gradient is generated within the Si electrode due to slow diffusion of Li through Si, which leads to localized strains. To reduce the interfacial stress and strain at 100% SOC, operation at moderate C-rates with low applied pressure is desirable. Alternatively, the mechanical properties of the SE could be tailored to optimize cell performance. To reduce Si stress, a SE with a moderate Young’s modulus similar to that of lithium phosphorous oxynitride (∼77 GPa) with a low yield strength comparable to sulfides (∼0.67 GPa) should be selected. However, if the reduction in SE stress is of greater concern, then a compliant Young’s modulus (∼29 GPa) with a moderate yield strength (1–3 GPa) should be targeted. This study emphasizes the need for SE material selection and the consideration of other cell components in order to optimize the performance of thin film solid-state batteries.

## 1 Introduction

In recent years, solid-state batteries (SSBs) have garnered significant attention from the academic research community and the electric vehicle and consumer electronics industries.^1−7^ The use of a solid electrolyte (SE) instead of the flammable liquid electrolyte used in conventional lithium-ion batteries (LIBs) can result in improved safety. Additionally, SSBs promise higher energy densities due to the pairing of the SE with a Li or Si negative electrode (NE). One of the main challenges associated with large format SSBs is their limited cycle life.^1,8−10^ The electrochemical and mechanical degradation at the solid–solid interfaces between the electrodes and SE is one of the causes of their rapid capacity deterioration.^11−19^

Si is more abundant than Li, is easier to manufacture roll-to-roll, and does not require moisture-free processing; therefore, it could be an alternative cost-effective NE to Li metal. However, this does not resolve the issue of electrochemical and mechanical degradation, which is augmented by its large volumetric expansion (as much as 300%) upon (de)lithiation. During cycling, these large volumetric changes can induce mechanical stresses within the SSB components, resulting in mechanical degradation, delamination, and fracture.^12,14,20,21^

Nevertheless, there is evidence^22,23^ that pairing an SE with Si can form a chemically stable interphase. This expands the portfolio of SEs available for use when paired with a Si electrode, in contrast with Li NEs for which there are very few that are chemically stable SEs. Sulfide SEs, for example, can be more easily integrated in a positive electrode composite (PE), forming a much lower impedance interface than oxide SEs. Furthermore, sulfide SE materials can be employed directly against Si, which would not be possible for a Li NE without the addition of coatings or buffer layers to prevent continuous decomposition.

Expanding the material design space would allow more SE materials^24,25^ to be used with potentially greater lifetimes (due to greater chemical stability against Si); a recent demonstration^23^ used a sulfide SE and achieved over 500 cycles.^23^ This study highlighted the importance of plastic deformation of the Li-Si alloy and applied stack pressure, both of which were thought to help maintain contact between the Si and SE. There are limited studies of SSBs that pair Si with conventional PE materials; therefore, there is a knowledge gap on how to improve these systems. The electrochemical parameters of these SSBs are crucial in validating accurate physics-based models that can help improve cell performance.^11,23,26−28^ Further, the sensitivity of SSB materials and mechanical parameters on rate performance as a function of applied pressure is poorly understood nor is the severity of the complex stress field generated during (de)alloying at the Si|SE interface.

Detailed electro–chemo–mechanical studies of SSBs commonly employ thin films,^11,29−31^ where the simple planar geometry, non-porous nature, high-rate capability and cycle life provide an excellent learning platform to better understand the complex interplay of the electrochemical and mechanical performance. These thin film SSBs are commercially available today, albeit with micro-Ah capacities. It is important to validate continuum scale models using such devices, which can be later extended to large-format SSBs as cycling data and parameters become increasingly available.

There is still debate in the literature on the desired SE mechanical properties for optimal cell performance when paired with a Si NE. Pioneering modeling work by Bucci et al.^32−34^ concluded softer and more compliant SEs (Young’s modulus <15 GPa) deform plastically and were more prone to microcracking.^32^ Conversely, other studies have argued that softer sulfur or solid polymer^35^ SEs exhibiting higher ductility help to alleviate stress in the NE, which is important for long cycle life.^22,23,36,37^ These studies modeled a composite electrode using a continuum framework but did not consider the full cell, and the results were not experimentally validated using full cell cycling data. A validated electro–chemo–mechanical model is desirable to investigate the internal stress field of thin film SSBs during realistic C-rates under applied pressure. To date, comprehensive, validated, continuum-level models are limited.

Our previous work^38^ demonstrated an experimentally validated electro–chemo–mechanical model of a Si thin film SSB. However, the electrochemical and mechanical interplay was not explored, nor was the dependency of externally applied pressure or C-rate. This work aims to understand the influence of these parameters to guide cell material design. The thin film SSB model consists of an amorphous Si (*a*-Si) NE, lithium phosphorous oxynitride (LiPON) SE and LiCoO_2_ (LCO) PE.^39,40^ First, the effect of SE mechanical properties and kinetics on first cycle efficiency are explored. Next, the applied pressure as a function of C-rate is investigated and a stress and strain map is presented. Finally, the effect of different SE material selection on the cell stress–strain response is discussed and used as a guide to lay out the desired SE mechanical properties for optimal cell performance. In several cases, the interfacial stress, concentration, and stress–strain gradients through the SSB domains are displayed and used to better understand the influence of applied pressure, C-rate, and electrochemical parameters on cell performance.

## 2 Model Formulation

**Figure 1:**
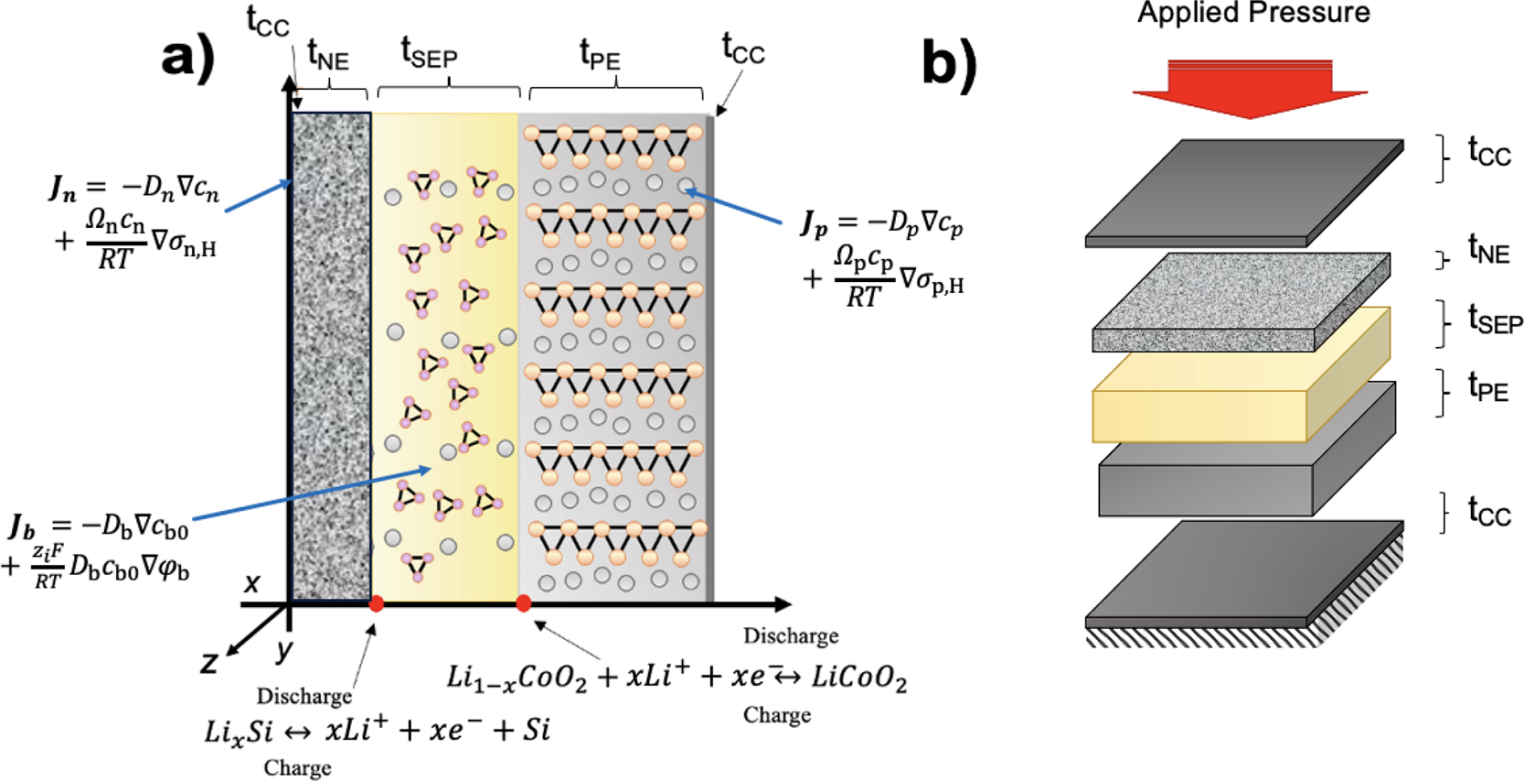
(a) 2D thin film SSB schematic, with the relevant electrochemical equations highlighted in each domain. (b) 3D SSB schematic displaying externally applied pressure to the top of the NE CC with the entire cell fixed at the bottom.

A schematic of the thin film SSB is depicted in Figure 1a, highlighting the reaction and solid-state transport equations in each domain. The schematic illustrates the current collectors (CCs), *a*-Si (NE), the SE separator, and LCO (PE) with only the SE being altered during the material design study. The thicknesses of the CCs, *a*-Si, SE, and LCO are represented as *t*_cc_, *t*_sep_, *t_ne_*, and *t_pe_*, respectively, while Figure 1b shows the applied pressure to the top of the cell and fully clamped conditions at the bottom of the cell, i.e., at the CC adjacent to the PE. An orthogonal coordinate system (Figure 1a) is used to define the thickness *x*, length *y*, and width *z* of the SSB. The electro–chemo–mechanical framework assumes a 2D geometry (given the film-like nature of the SSB). Li transport occurs in 1D (*x*–*y* plane), while deformation is assumed to be plane strain. It is acknowledged that most of the deformation, which is large in magnitude, occurs in the *x* direction that leads to a significant degree of anisotropy. This work assumes isotropic material properties due to lack of anisotropic values, and it is encouraged for researchers to better evaluate these. To reduce computational demand, a small subvolume of the overall cell thickness *x* was modeled and assumed to be representative of the cell and applied symmetric boundary conditions. We note that the model assumes perfectly conformal interfaces, as approximated in devices fabricated by vacuum deposition techniques. However, in devices where surfaces which have simply been physically contacted, conformal contact cannot be assumed hence contact area in these types of devices should be carefully considered and appropriately modeled.

### 2.1 Material Parameters and Boundary Conditions

For the baseline case, the cell configuration was *a*-Si NE, LiPON SE, and LCO PE. In this work, the SE material was varied, and the mechanical and electrochemical parameters used are recorded in Table 1. Here, the ionic conductivity, elastic modulus, and yield strength are denoted as *K*, *E*, and σ_Y_, respectively, with the subscript relating to the type of SE material. The chosen sulfide and oxide SE materials were Li_6_PS_5_Cl (LPSCl) and Li_6.4_La_3_Zr_1.4_Ta_0.6_O_12_ (LLZTO), respectively. Both are commonly used SEs with relatively high ionic conductivities, and their mechanical properties and hardness have been characterized by Papakyriakou et al.,^41^ where LPSCl and LLZTO were found to exhibit viscoplastic creep behavior with a creep rate coefficient, *B* (s^–1^) and stress exponent, *n_SE_* (Table 1). The yield strength is calculated via Tabor’s relationship where it is equal to a third of the hardness (MPa).^42^ It is acknowledged that hardness measurements can lead to a variation in results, which would translate into the calculate yield strength; however, as direct yield strength measurements are lacking in the literature, the use of hardness values was justified in this case. It is suggested for researchers to investigate the nature of fracture and post-yield behavior of SE materials. A Chaboche-type viscoplasticity^43^ model is used herein, where the reference stress is set equal to the SE yield stress as the reference stress values for these SE materials are not known. However, this model is an approximation since the experimental post-yield stress–strain response of these SE materials has not yet been reported to the best of our knowledge.

**Table 1:** Solid Electrolyte Material Parameters

			parameter	units	value	source
electrochemical			*K_*_LiPON_	S cm^–1^	2.3 × 10^–6^	ref (44)
			*K_*_LPSCl_	S cm^–1^	2.9 × 10^–3^	ref (41)
			*K_*_LLZTO_	S cm^–1^	5.9 × 10^–4^	ref (41)
elastic			*E_*_LiPON_	GPa	77	ref (45)
			*E_*_LPSCl_	GPa	29	ref (41)
			*E_*_LLZTO_	GPa	125	ref (41)
plastic			σ_*Y_*LiPON_	GPa	1.33	ref (45)
			σ_Y_LPSCl_	GPa	0.67	ref (41)
			σ_Y_LLZTO_	GPa	3	ref (41)
			*n_*_LPSCl_	1	20	ref (41)
			*n_*_LLZTO_	1	45	ref (41)
			*B_*_LPSCl_	s^–1^	6 × 10^–4^	ref (41)
			*B_*_LLZTO_	s^–1^	1 × 10^–4^	ref (41)

Treatment of the LiPON SE was extended from an isotropic linear-elastic solid to include plastic deformation. In the absence of mechanical studies on its post-yield behavior, LiPON was modeled as a perfectly plastic solid. LPSCl and LLZTO were also modeled as elastic-perfectly plastic solids. LCO was assumed to be an isotropic linear-elastic solid, whereas *a*-Si was treated as an isotropic elastic-viscoplastic solid with its Young’s modulus, yield strength, and Poisson’s ratio varying with the state of lithiation (further details can be found in Vadhva et al.^38^ and Leo et al.^46^). The CCs were assumed to be electronically conductive, linear elastic solids with Young’s moduli of ≈100 GPa. Finally, the universal gas constant, *R*, was taken as 8.314 J mol^–1^ K^–1^, and all simulations were conducted at a temperature, *T*, of 298 K.

Various boundary conditions were considered to probe the influence of pressure and constraint on electrode behavior and are outlined as follows:

1. An applied pressure at the CC adjacent to the NE electrode in the range of 0–500 MPa. A fully clamped constraint (zero displacements in *x*, *y*, and *z*) at the CC adjacent to the PE was applied (for all simulations up to Figure 7).
2. In a separate study, fully clamped conditions were applied at both CCs to assess the maximum level of constraint (for Figures 8–10).

It is acknowledged that the pressure range used in (1) is generally higher than that used in lab settings (up to ∼250 MPa^47^); however, to show a clear trend between the applied pressure, C-rate, and averaged cell capacity, the simulated pressure was modeled up to 500 MPa. While these pressures may be feasible for small scale laboratory and thin film solid-state cells, it should be noted that for the commercialization of large-format SSBs with a cell areas ∼3–4 orders of magnitude greater, even an applied pressure of 100 MPa will be very challenging, and researchers should be aware of this when designing large-format SSBs.

### 2.2 Simulation Details

The electro–chemo–mechanical model was created using the finite element modeling software package, COMSOL Multiphysics (v6.0 Sweden). The 2D mesh consisted of approximately 4000 quadratic elements with 94,000 degrees of freedom, while the solutions were found to be mesh-independent. The Parallel Direct Sparse Solver (PARDISO) was used to solve the discretized transport, electrochemistry, and solid mechanics equations, using the numerical procedure previously outlined.^38^

## 3 Results and Discussion

### 3.1 First Cycle Efficiency

**Figure 2:**
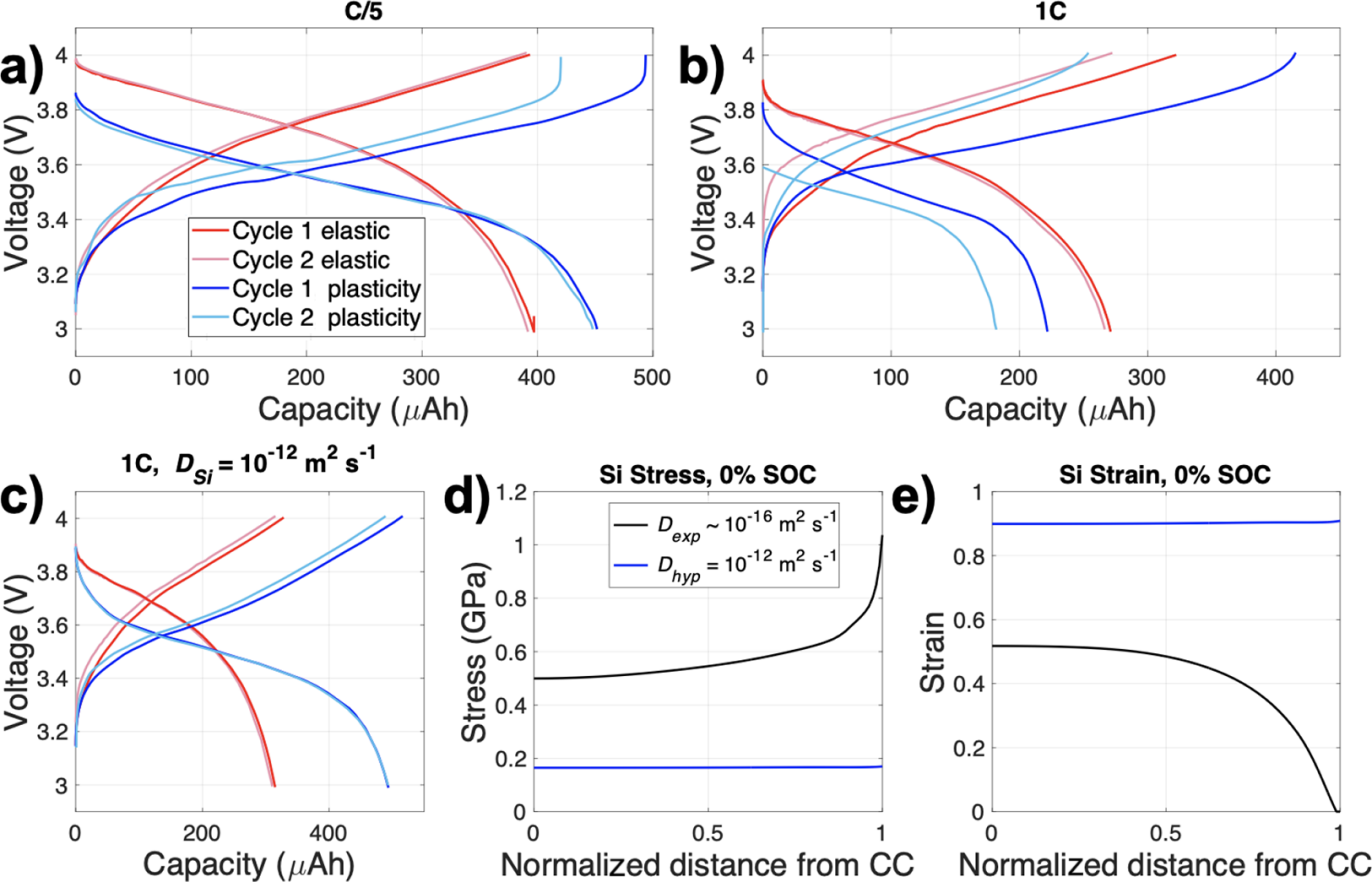
(a) C/5 voltage profile considering Si elastic only behavior (red) and with the inclusion of Si plasticity (blue). The darker shade indicates the first cycle, whereas the lighter shade represents the second cycle. (b) Voltage profile at 1C and (c) with a higher Li diffusion in Si (*D_hyp_* = 10^–12^ m^2^ s^–1^). (d) Si nominal stress and (e) nominal strain at 0% SOC, using *D_exp_* ∼ 10^–16^ m^2^ s^–1^ (black) and *D_hyp_* = 10^–12^ m^2^ s^–1^ (blue). The legend in panel (a) applies to panels (a) to (c) while the legend in panel (d) applies to panels (d) and (e).

Under a C/5 rate, charge–discharge of cycles 1 and 2 of the baseline cell were simulated (Figure 2a). Two different cases were modeled: (1) Si as a linear elastic solid (elastic behavior only, red lines) and (2) Si as a viscoplastic solid (plastic behavior included into the model, blue lines). There was a pronounced difference in the charge capacities between cycle 1 and 2 for the Si plasticity case and a much smaller difference for the Si elastic case. This points to Si plastic deformation occurring during the first cycle that caused changes in electrode response from the start of charge to the end of discharge, resulting in reduced cell capacity. This observation is in agreement with experimental findings by Han et al.,^48^ who suggested that the difference in the first and second cycle capacity may be due to Si–Si bond breaking and plastic deformation during the first lithiation. It is important to note that the cell capacity increased with the inclusion of Si plasticity given that the plastic deformation of Si reduces the build-up of stress. The reduction in lithiation-induced stress overpotential reduces the overall cell overpotential, allowing further lithiation before the upper voltage limit is reached.

Increasing the rate to 1C (Figure 2b) while modeling Si as an elastic material resulted in greater first cycle capacity reduction when compared to C/5 cycling, albeit less than when Si plasticity was considered. To understand the cause of this phenomenon, the diffusion of Li in Si, as taken from experimentally extracted diffusion coefficient in the literature (*D_exp_* ∼ 10^–16^ m^2^ s^–1^)^38^ was increased by a factor of 2, which still resulted in a large discrepancy in capacity retention as can be seen in the Supplementary Information (SI) Figure S1. As the Li diffusion in Si was progressively increased from a factor of 2 to ∼4 orders of magnitude, there was a progressive increase in the capacity retention and only at ∼4 orders of magnitude with a hypothetical diffusion coefficient of *D_hyp_* = 10^–12^ m^2^ s^–1^ was the capacity difference between cycles minimal (Figure 2c and clearly contrasted in Figure S1). It is acknowledged that increasing the Li diffusion coefficient in Si by ∼4 orders of magnitude could also introduce other artifacts such as excessive plastic deformation leading to capacity loss via the stress induced overpotentials. However, this is ruled out by modeling both the elastic and plastic cases, which showed the same capacity between cycles (Figure 2c); therefore, the Li diffusion in Si is the main factor behind the capacity difference. It must also be noted, by extension, that the electrode thickness will influence the extent of reduction in first cycle capacity given the transport limitations that are typically associated with thicker electrodes.^49^

Herein, we denote 100% and 0% state of charge (SOC) as the end of charge and end of discharge of the first cycle respectively. It should be noted that the stress and strain outlined in this study are always the nominal principal stress and strain. Considering the Si stress and strain at 0% SOC, a much lower tensile stress (Figure 2d) was exhibited for the higher diffusion coefficient of 10^–12^ m^2^ s^–1^ compared to the experimentally extracted diffusion coefficient (*D_exp_*), which is of the order ∼10^–16^ m^2^ s^–1^ depending on the SOC. The observed Si strain was lower than the theoretical maximum strain of 3 (300% volumetric expansion) under the cell voltage cycling limits given that full Si lithiation did not occur. As the extent that Si is lithiated is governed by the Li inventory from LCO, the thickness of the LCO dictated the maximum possible lithiation of Si. When normalizing the Si concentration gradient, it was the maximum amount of Si lithiation that was achieved during cycling that was used. Experimental evidence of partial Si lithiation was observed previously using differential capacity analysis for these cells.^38^

The Si strain was considerably greater when the higher diffusion coefficient was implemented (Figure 2e) due to increased Si lithiation, which resulted in greater strains but also increased cell capacity. In addition, the Si strain was more homogeneous through the electrode than with the slower experimental diffusion coefficient (Figure 2e), which can be directly linked to the Li concentration gradients throughout the electrode due to diffusion-related transport limitations. Further, for the Si plasticity case with a faster diffusion coefficient (Figure 2c), a much higher first cycle discharge capacity at 1C (∼ 450 μAh) was displayed than with the experimentally extracted diffusion coefficient (∼ 225 μAh in Figure 2b). The capacity difference between cycles was not as pronounced for the Si elastic case. Therefore, the Li diffusion rate in Si, thickness and the mechanical properties of Si greatly influence the achievable cell capacity and stress–strain response.

### 3.2 Applied Pressure

To understand the influence of applied pressure on the electrochemical performance of the baseline SSB, a pressure of 500 MPa was applied. Two C-rates (1C and 5C) were simulated under the applied pressure to observe the evolution of stress and strain in the Si NE. The higher C-rates were chosen to observe how the larger concentration gradients in the Si affected the local stress–strain response. Additionally, the Si stress at 0% SOC under an applied pressure of 500 MPa was compared to the zero-pressure condition for both C-rates. There was minimal change in the stress and strain of LCO during cycling (strain ∼2% in LCO^50^), hence, the stress and strain generated in the NE and SE during cycling was primarily considered.

**Figure 3:**
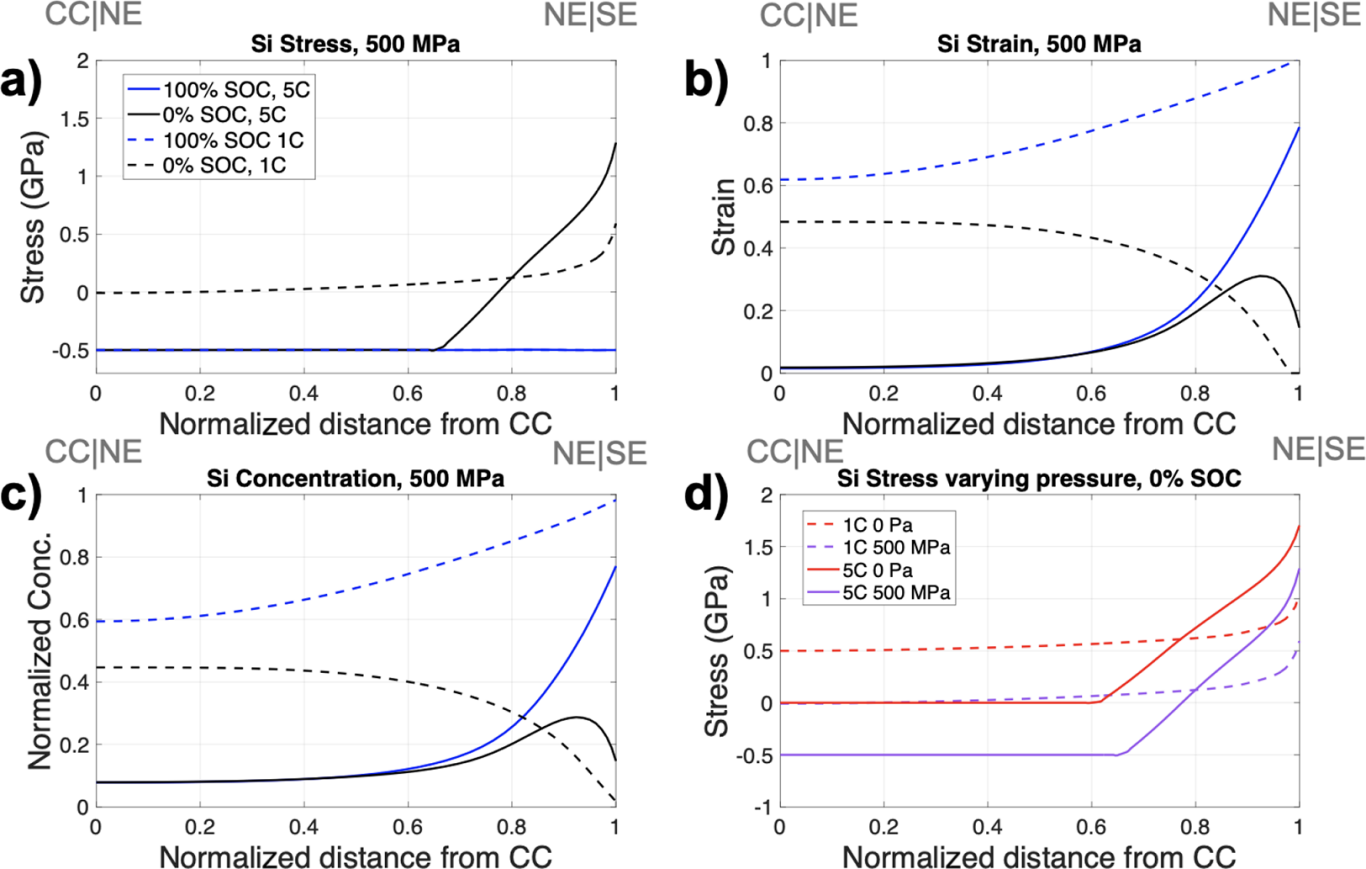
Results simulated under 500 MPa applied pressure at 5C (solid line) and 1C (dashed line) at 100% SOC (blue) and 0% SOC (black) showing (a) nominal stress in Si, (b) nominal strain in Si, (c) normalized Si concentration with respect to the maximum acceptable concentration, and (d) nominal stress in Si at 0% SOC with varying pressure of 0 MPa (red) and 500 MPa (purple) at 1C (dashed) and 5C (solid line). The legend displayed in panel (a) applies to panels (a)–(c) with the gray text on top of the figures indicating the cell configuration (e.g., the CC and NE interface (CC|NE) at 0 normalized distance from the CC).

Figure 3a shows that at 0% SOC (end of discharge), a larger tensile stress was observed for 5C (black solid line), which occurred near the Si|SE interface, moving to compressive stress further into the Si electrode, toward the CC. This is due to the concentration gradient that is present during discharge and is related to the sluggish solid-state transport of Li ions in Si. This means that toward the Si|SE interface, more Li is removed (reducing the stress) but further into the electrode some Li remains, which produces a lithiation-induced compressive stress. The Si stress for the 1C case at 0% SOC (dashed black line) was more homogeneous throughout the electrode with lower tensile stress at the Si|SE interface and a gradual decrease in stress to zero (no compressive stress observed). At 100% SOC, the Si stress at both C-rates was the same.

The Si strain profile (Figure 3b) is analogous to the state-of-lithiation in Si, (Figure 3c, normalized with respect to its maximum concentration) because the strain occurs due to the lithiation of Si. At the end of discharge for the 5C case, a local peak in Li concentration and strain occurred at ∼0.9 normalized distance from the CC. This peak is associated with transport limitations due to slow Li diffusion in Si. After charge at 100% SOC, the material adjacent to the NE|SE interface has reached a high level of lithiation (∼0.8), while there is a gradient in concentration, with the degree of lithiation reducing toward the CC. Upon discharge, we see similar non-linear delithiation behavior, where the material closest to the SE sees a larger extent of extraction. Most of the extracted Li was from the highly lithiated region (greater than 0.9 distance from the CC) as a consequence of charging – slow diffusion means that not all of it could be extracted in a homogeneous manner, especially in regions close to the CC, resulting in the localized peak at 0% SOC. This leads to localized strains at that distance into the electrode. The concentration and strains are highest at 100% SOC due to maximum lithiation and are higher for 1C (dashed blue line) than 5C (solid blue line) due to the slower C-rate allowing greater lithiation. The concentration and strain profiles during 1C cycling were more homogeneous throughout the Si than in the 5C case.

Figure 3d compares the electrode response with and without applied pressure. The Si stress at 0% SOC highlights the non-linear stress response: for 1C at zero applied pressure (dashed red line), Si exhibits tensile stress toward the CC as a result of delithiation, with a gradual increase in stress due to lower Li content toward the Si|SE interface. At 500 MPa (dashed purple line) however, there is compressive stress due to the pressure that is applied. This external pressure counteracts the tensile stress within the Si electrode due to delithiation and results in an almost stress-free state at the CC, with increasing tensile stress further into the electrode. For the 5C case with no applied pressure (solid red line), there are low concentrations of Li at the CC, causing low, or close to zero stress at this location, with increasing tensile stress through the remainder of the electrode, similar to the 1C case. For the cell simulated at 5C with 500 MPa pressure (solid pink line), the CC region experiences commensurate compressive stress, with tensile stresses developing toward the interface due to high levels of lithiation close to the SE.

**Figure 4:**
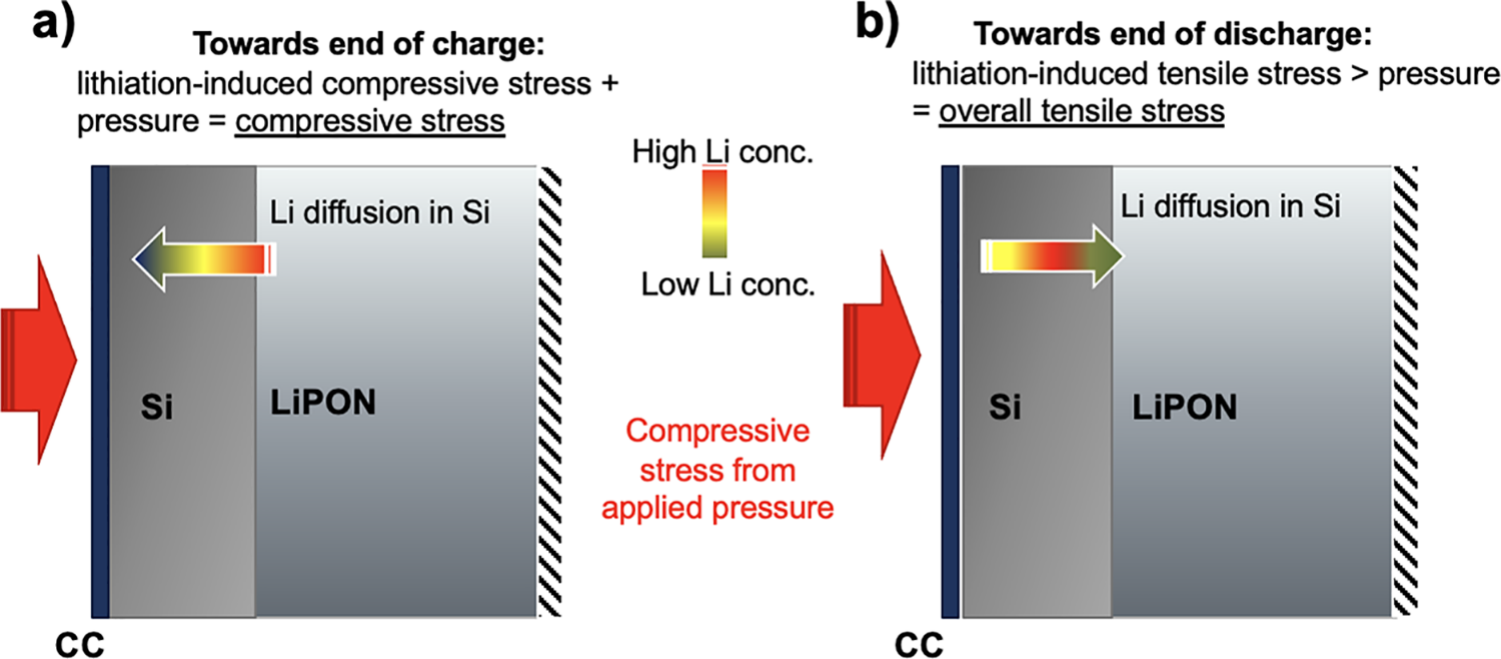
Schematic of compressive applied pressure and lithiation-induced stress toward (a) the end of charge and (b) the end of discharge. The Li concentration gradient is represented by the arrow’s color gradient with the solid red arrows representing the compressive stress from the applied pressure. The LCO and bottom CC are omitted for clarity, with the dashed black lines representing that the bottom of the cell is fixed.

To help visualize the compressive applied pressure stress and lithiation-induced stress, which is compressive during charge and tensile during discharge, the schematic is presented (Figure 4a,b). The buildup of Li concentration gradients within the Si toward end of charge (Figure 4a) and end of discharge (Figure 4b) is displayed and used to understand the non-linear stress and strain response displayed in Figure 3.

**Figure 5:**
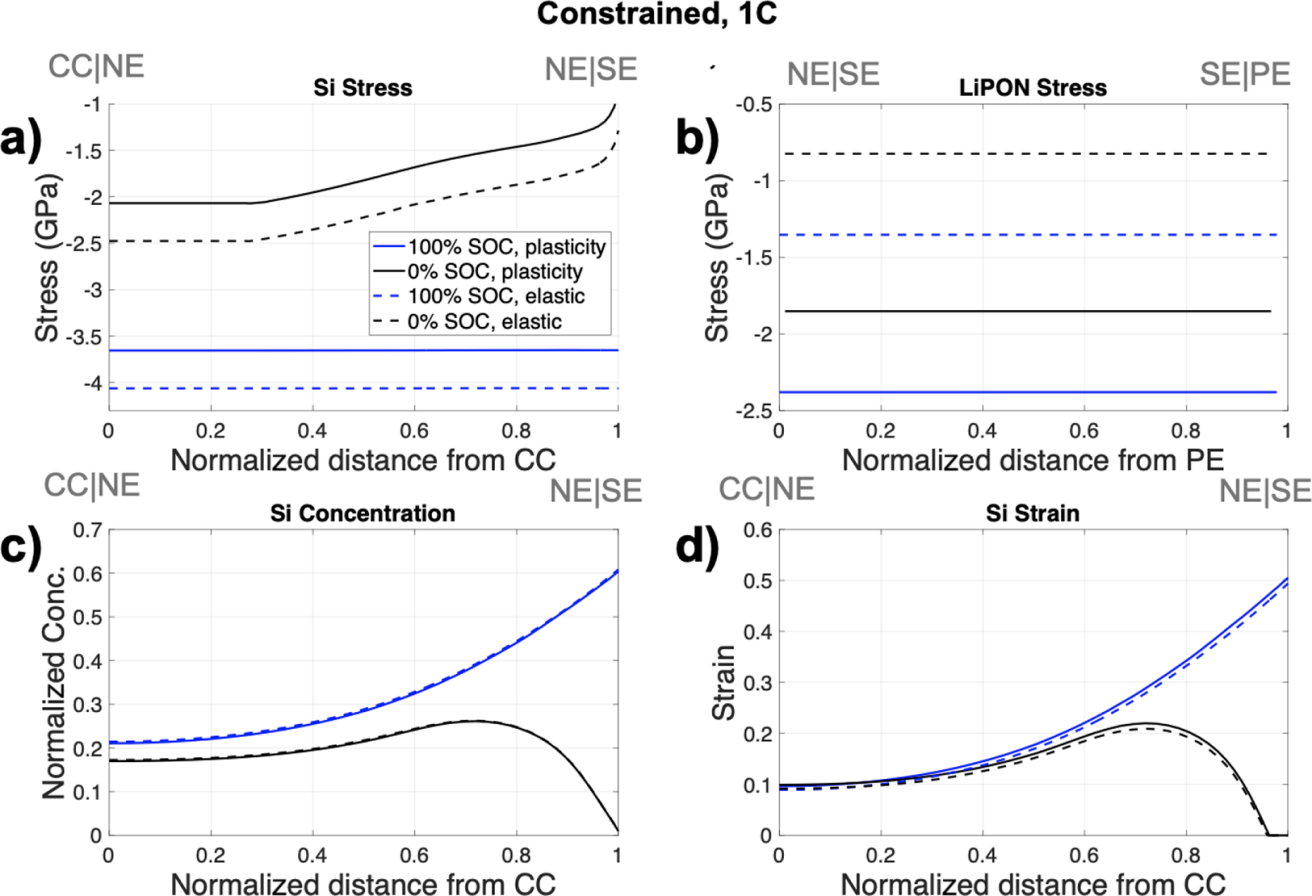
Constrained case simulated for LiPON plasticity (solid line) and elastic behavior only (dashed line) at 100% SOC (blue) and 0% SOC (black) showing (a) Si stress, (b) LiPON stress, (c) Si concentration, and (d) Si strain. The legend displayed in panel (a) applies to all figures with the gray text on top of the figures indicating the cell configuration.

Under the applied pressure case of 500 MPa, the yield strength of LiPON (1.33 GPa in Table 1) was not reached. To explore the effect of LiPON plastic deformation, the cell was simulated as fully constrained to guarantee yielding and cycled at a 1C rate. In this study, the influence of LiPON material response was considered; a comparison was drawn between an elastic material and of an elastic-perfectly plastic response. The Si stress reduced when LiPON plasticity (solid lines) was considered (Figure 5a) at 100% and 0% SOC while the stress in LiPON increased as a result of plastic deformation for the fully constrained case (Figure 5b). The concentration and Si strain remain very similar for both cases (Figure 5c,d).

**Figure 6:**
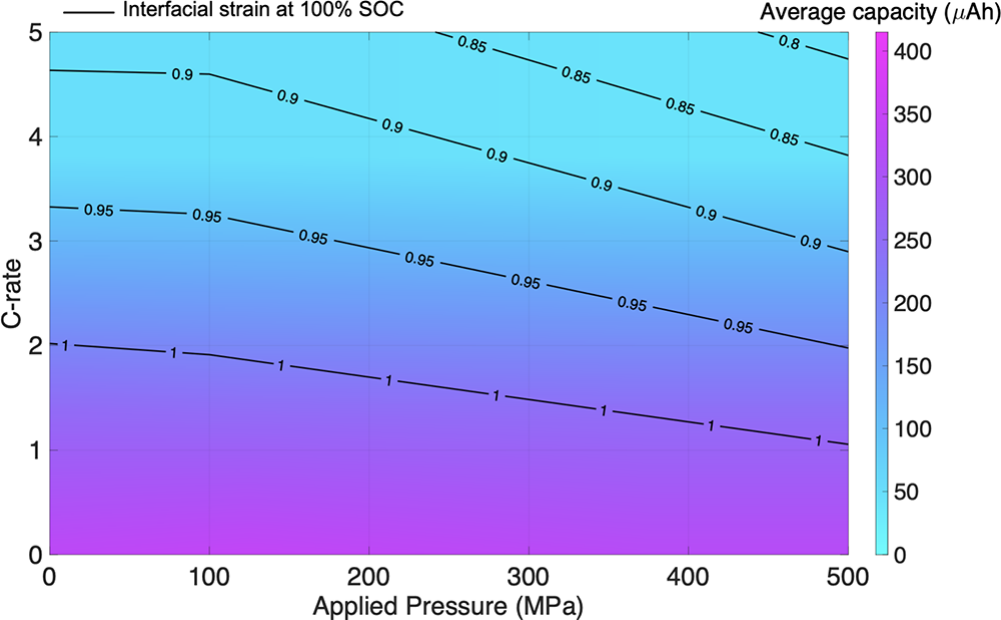
Contour map of maximum principal strain at 100% SOC (solid black lines) at the Si|LiPON interface as a function of C-rate versus applied pressure, with corresponding-colored contours of average cell capacity.

**Figure 7:**
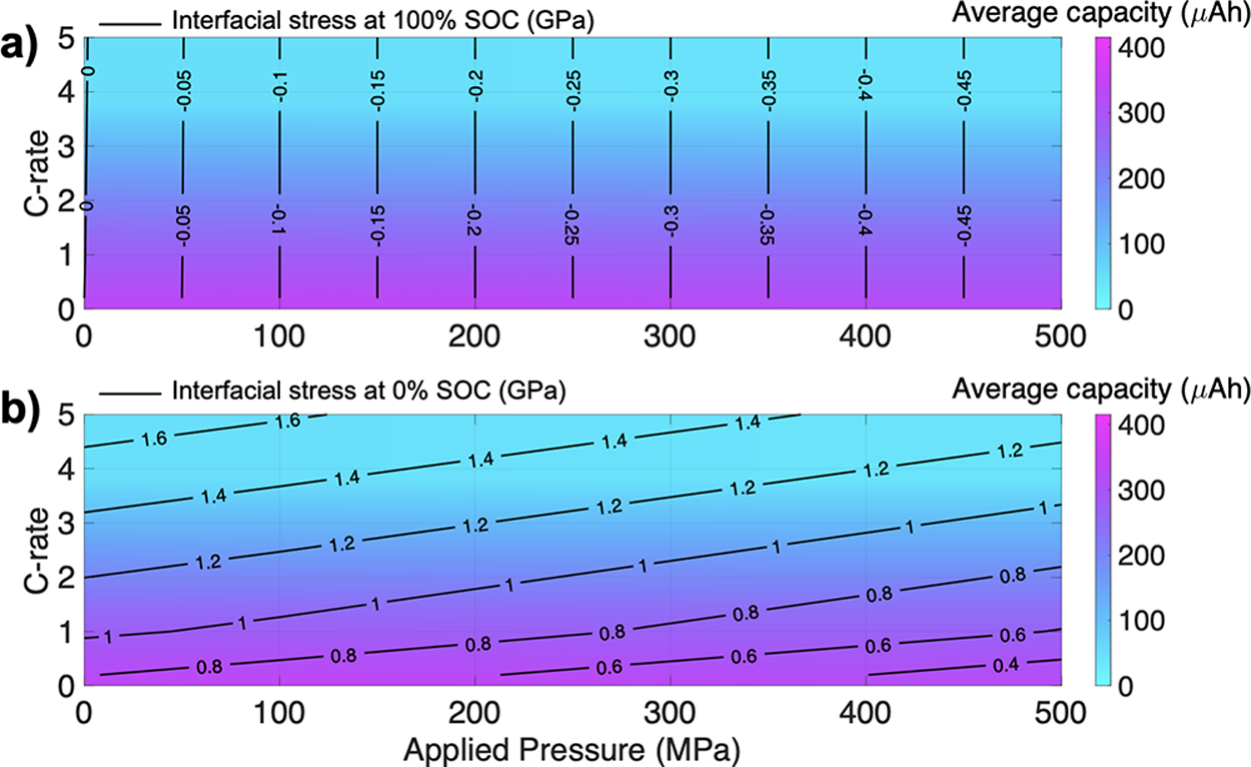
Contour map of maximum principal stress in GPa (solid black lines) at the Si|LiPON interface as a function of C-rate versus applied pressure with corresponding-colored contours of average cell capacity at (a) 100% SOC and (b) 0% SOC.

To understand the effect of applied pressure and C-rate on the cell capacity, a map was generated where five C-rates under five pressure values were simulated, with intermediate values determined by linear interpolation. Figure 6 shows a map of the maximum principal strains that occurred at the Si|LiPON interface. As the C-rate increased, the stress overpotential became larger, reaching the maximum cell voltage quicker, thereby reducing the overall capacity. Note that the average capacity discussed in this section and in Figures 6 and 7 is an average of the cell’s charge and discharge capacities. As the C-rate increases, the lower degree of lithiation also reduces the maximum principal strain (black contours in Figure 6). For a given C-rate, there is little change in capacity as the applied pressure is increased, highlighting the cell strain response is more sensitive to the C-rate than the applied pressure in the range of 0–500 MPa. This is in line with our previous findings (Figure 3b), which showed the driver of localized strains within the Si is the higher C-rate (5C versus 1C). However, when simulated under extreme and highly unrealistic stack pressures, a pressure dependency was seen on the cell capacity (applied pressure > 3 GPa, seen in Figure S2).

The stress at 100% SOC (black contours in Figure 7a) is significant in that as the applied pressure increased, the compressive stress also increased, but the stress was independent of C-rate. Again, the average cell capacity is largely dependent on C-rate and minimally affected by applied pressure. At higher C-rates, the cell capacity was reduced due to slow Li ion diffusion in Si, which produced non-linear concentration gradients within the electrode and reduced the degree of lithiation. The stress experienced at the interface remains compressive for all non-zero applied pressures. By contrast, the stress at 0% SOC (Figure 7b) depends both on C-rate and applied pressure. The stress and strain experienced at the interface are due to the tensile lithiation-induced stress during discharge. As the C-rate was increased, the tensile stress also increased and the buildup of stress under these conditions could be of concern for void formation as well as possible Si and/or SE fracture. It is interesting to note at a given C-rate that, as the applied pressure increases, the stress is reduced due to the applied pressure, which exerts a compressive force (clearly visualized in Figure 4b), resulting in an overall reduced tensile stress. The capacity is influenced by the C-rate, reducing at higher C-rates, with little dependence on applied pressure.

### 3.3 Solid Electrolyte Material Selection

To explore the mechanical properties of the SE and its influence on the resulting stress and strain, the SSB was extended from the baseline case (LiPON SE) to include other SEs. Two commonly used SEs were chosen, both of which display different electrochemical and mechanical properties (outlined in Table 1). In all cases, the SSB was fully constrained to probe the effect of SE plastic deformation.

**Figure 8:**
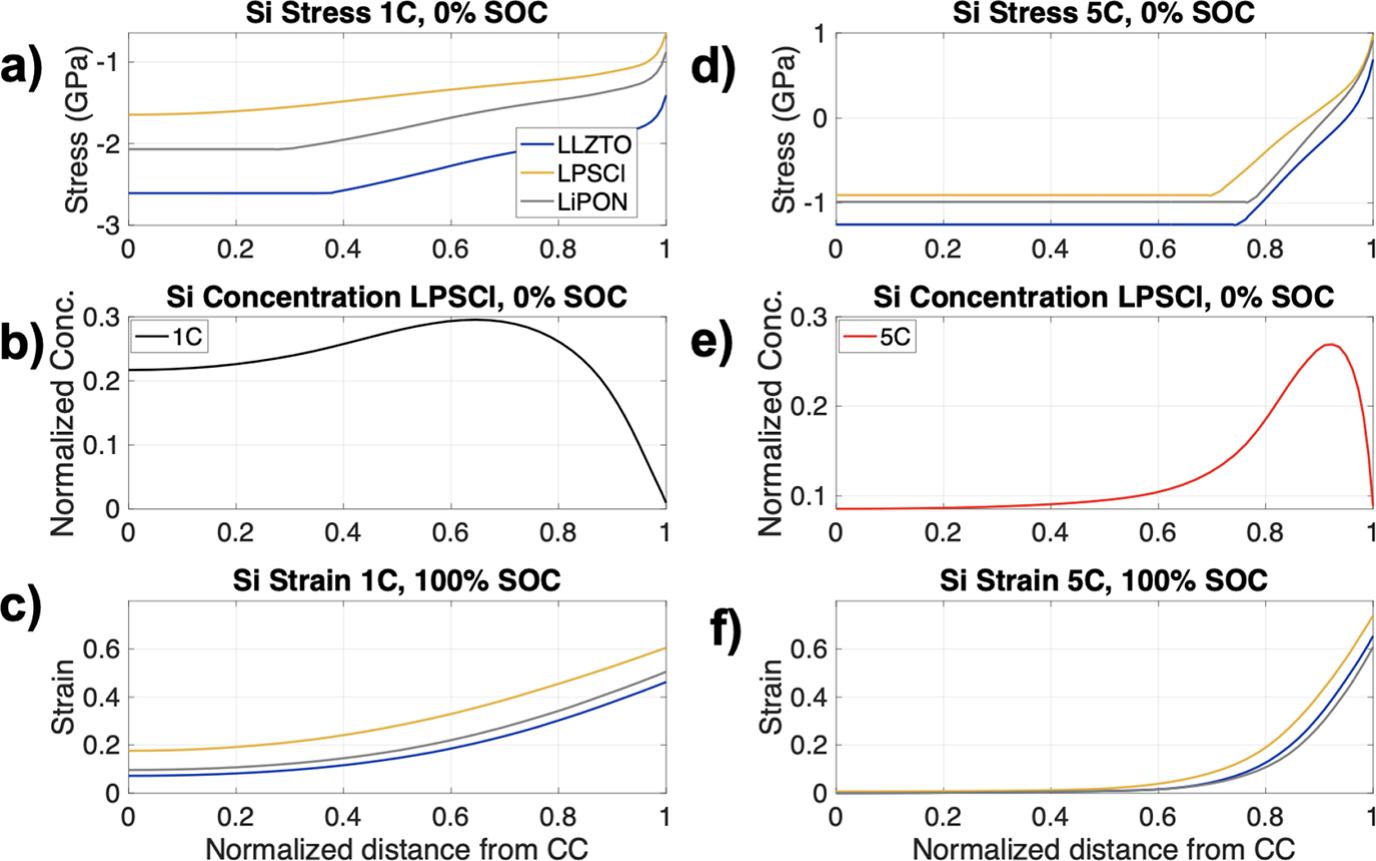
Si stress at 0% SOC at (a) 1C and (b) 5C for the three different SEs: LPSCl (yellow), LiPON (gray), and LLZTO (blue). The Si concentration for the LPSCl case at 0% SOC at (c) 1C (black) and (d) 5C (red). The Si strain at 100% SOC is displayed for (e) 1C and (f) 5C. Legend in panel (a) applies to panels (a), (c), (d), and (f) while panels (b) and (e) have their own legends.

The Si stress at 1C, 0% SOC was non-linear for all SEs (Figure 8a); however, compared to LiPON, the Si stress increased for the LLZTO case but reduced for the LPSCl case. At 5C, the spread in Si stress reduced for the three different materials (Figure 8b), highlighting the importance of kinetics on the Si stress response. The stress at 5C was observed to change from compressive (at the CC) to tensile at the Si|SE boundary. In contrast, at 1C the stress remained compressive through the Si, though it reduced in value toward the Si|SE interface. The reason behind the tensile behavior at 5C can be understood by analyzing the different concentration gradients within the Si NE for the LPSCl case at 1C (Figure 8c) versus 5C (Figure 8d). The Li concentration in Si at 5C is highly non-linear exhibiting a turning point in the concentration gradient due to the slow diffusion of Li in Si, at a normalized distance from the CC (∼0.9) and a subsequent drop in concentration. This rapid reduction in concentration likely reduced the stress at the interface, and since the top of the cell is constrained, a mix of compressive stress and tensile stress exists within the Si. By comparison, the concentration gradient in the Si at 1C (Figure 8c) showed a gradual decay in concentration. Only the Si concentration gradient for the LPSCl case is modeled here for clarity as the profiles follow a similar trend for the other two SEs.

**Figure 9:**
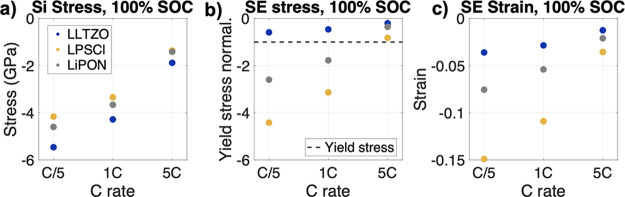
(a) Si stress at 100% SOC for the three different SEs: LPSCl (yellow), LiPON (gray), and LLZTO (blue) at C/5, 1C, and 5C. (b) The SE stress normalized by its yield stress (dashed line) and (c) SE strain at 100% SOC. The legend in panel (a) applies to all figures.

The Si strain at 100% SOC (Figure 8e) was highest for the LPSCl case, which was expected as it exhibited the lowest stress and therefore highest Si lithiation. Although LPSCl exhibited the highest strains (Figure 9c), which could be undesirable from an engineering standpoint, it also had the highest amount of lithiation, which will result in the greatest cell capacity. The spread in strains was larger for 1C versus 5C for the different SE materials (Figure 8e,f) and the stress–strain profiles were more homogeneous throughout the Si electrode for 1C.

At 100% SOC, the Si stress and SE stress and strains at different C-rates (C/5, 1C, and 5C) were constant throughout the domains; therefore, a scatter plot was chosen to best represent the results (Figure 9). The Si stress (Figure 9a) was greatest for the LLZTO case but the stress (Figure 9b) and strain (Figure 9c) within LLZTO were significantly lower compared to LiPON and LPSCl. LLZTO did not yield under any C-rate and as a result displayed lower stress and strain. LPSCl has the lowest yield strength, which resulted in the early onset of plastic deformation, the Si stress was reduced. This resulted in increased lithiation, which means it experienced the highest SE stress and strain for all C-rates. This could be concerning for low yield strength materials such as LPSCl, which deformed by as much as 15% even at the low C-rate condition of C/5. As previously observed, as the C-rate increased, the spread in stress–strain values between the three materials decreased.

### 3.4 Solid Electrolyte Design for Optimal Cell Performance

**Figure 10:**
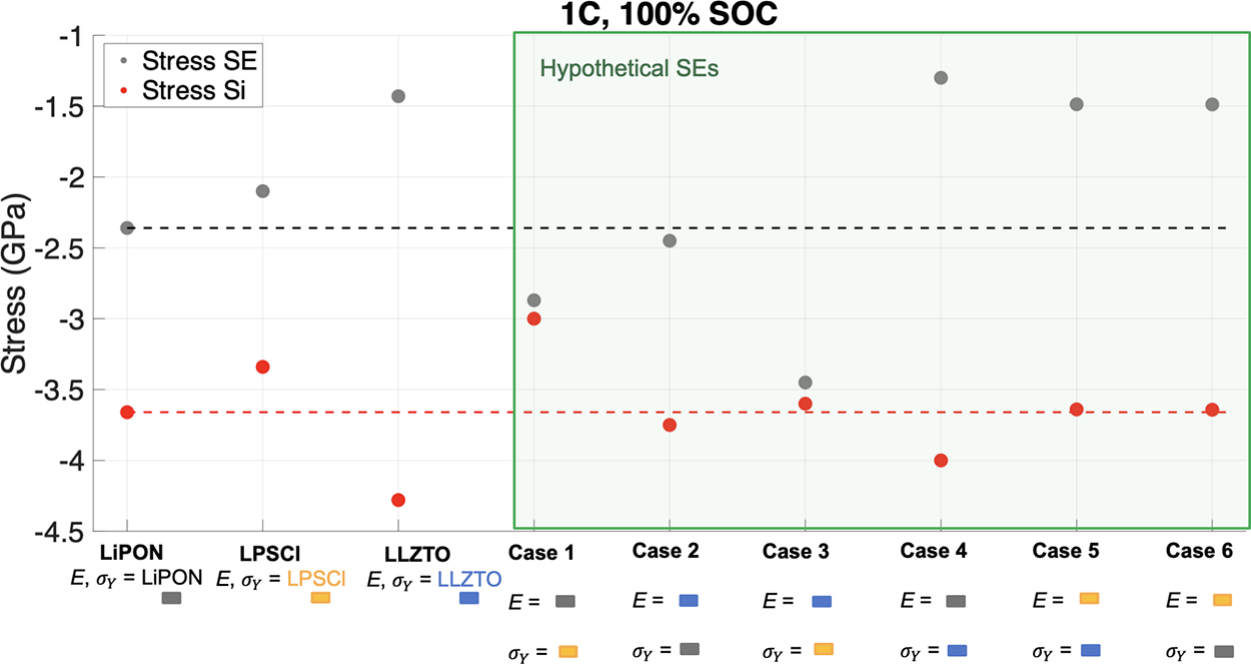
Si (gray dots) and SE (red dots) stress response at 1C, 100% SOC. LiPON, LPSCl, and LLZTO SEs are contrasted against six simulated hypothetical SEs with Young’s modulus and yield strength values taken alternatively from LiPON, LPSCl, and LLZTO, which are represented by colored rectangle symbols (gray for LiPON, yellow for LPSCl, and blue for LLZTO). The dashed line across the *y* axis highlights the LiPON SE and Si stress values from the baseline study.

This section considers the SE mechanical properties for optimal cell performance under the fully constrained case at 1C, 100% SOC. Following the discussion on the importance of the SE mechanical properties on the stress–strain response, six cases of hypothetical SEs were simulated with Young’s moduli and yield strength values taken alternatively from LiPON, LLZTO, and LPSCl (Figure 10). Such materials could be realized via composites or SE material discovery. For comparison, LiPON, LLZTO, and LPSCl are displayed alongside the different SE cases, with the LiPON SE (gray dashed line) and Si (red dashed line) stress values displayed for reference.

In case 1, Young’s modulus of LiPON was chosen and paired with a lower yield strength equal to LPSCl. This resulted in a reduction in Si stress compared with LiPON due to the earlier onset of the SE yielding and subsequently increased the SE stress. Case 2 simulates Young’s modulus of LLZTO with a moderate yield strength equal to that of LiPON. Compared to pure LiPON, a minimal increase in the SE and Si stress occurred. In case 3, the SE stress increased significantly (∼1.1 GPa) due to the lower yield strength, while Si stress reduced minimally (∼0.1 GPa). Case 4 simulated Young’s modulus of LiPON with relatively higher yield strength equal to that of LLZTO. The SE stress reduced significantly (∼ −1 GPa) as the material yield onset was delayed which increased the Si stress (∼0.35 GPa). For cases 5 and 6, LPSCl Young’s modulus was chosen with a yield strength equal to LLZTO and LiPON, respectively. There was little change between the two cases, though case 6 has a slightly lower yield strength, which increased the SE stress and reduced the Si stress minimally. Compared to the LPSCl SE, cases 5 and 6 show an increased Si stress (∼0.2 GPa) but with a greater reduction in SE stress (∼0.6 GPa) due to the higher yield strength.

Overall, a trend was observed whereby choosing a relatively moderate Young’s modulus, similar to that of LiPON, helps to reduce the Si stress and pairing with a low yield strength allows for the early onset of SE yielding which further reduces Si stress (case 1) but results in an increase of SE stress. Selecting a relatively low Young’s modulus material such as LPSCl with a high or moderate yield strength (case 5 or 6) reduces the SE stress similar to case 4. However, in comparison to case 4, the Si stress is reduced further by ∼0.4 GPa. If reducing the stress in the Si NE is of primary concern, then an SE with a moderate Young’s modulus and low yield strength (case 1) should be chosen. If, however, a reduction in SE is of greater importance, then as cases 5 or 6 show, a low Young’s modulus and high or moderate yield strength should be adopted. Selecting a high Young’s modulus similar to LLZTO did not provide much benefit against the baseline LiPON case in either Si or SE stress. If a high Young’s modulus material is to be used, then its advantage is in its superior yield strength, which reduces the SE stress, although a greater reduction in both SE and Si stress can be achieved with a moderate Young’s modulus similar to that of LiPON tailored with the high yield strength of LLZTO. Therefore, a high Young’s modulus alone is not advantageous in reducing the Si and SE stress – the yield strength also plays an important role.

## 4 Conclusions

A previously validated electro–chemo–mechanical model of a thin film SSB with a Si NE^38^ was used to understand the effect of mechanical and electrochemical properties on the first and second charge–discharge cycles. Then, the effect of applied pressure and C-rate on the average cell capacity and stress–strain response was probed. Finally, the mechanical properties of the SE were tailored for minimal interfacial stress and strain. Key insights include

(1) Modeling Si plasticity and the diffusion of Li ions in Si greatly influences the achievable first cycle capacity. Focus should be drawn to the mechanical and electrochemical parameters of Si when optimizing SSB cycle life.
(2) The interfacial stress at 100% SOC was found to be C-rate independent and increased as a function of applied pressure. Thus, to reduce the interfacial stress and strains at 100% SOC and increase cell capacity, low to moderate C-rates (1–1.5C) and applied pressure are desirable (< 200 MPa).
(3) The stress experienced at the end of discharge was tensile, which is of concern as it could lead to void formation during discharge. To reduce the interfacial stress at the end of discharge and increase cell capacity, low C-rates (<1C) and moderate applied pressure (100–200 MPa) are desirable as the applied pressure reduces the overall tensile stress.
(4) The capacity was strongly influenced by the C-rate and minimally affected by applied pressure. As the C-rate increased, the average capacity reduced greatly (factor of ∼5 from 1C to 5C, with no applied pressure). This emphasizes that the slow Li ion diffusion in Si is a key driver of the localized concentration gradients and limits the achievable cell capacity. Strategies to mitigate this aspect include use of a thinner Si electrode with added porosity^23^ to reduce the Li-ion diffusion path length, which is conducive to Li insertion/extraction into the Si or nano-structuring of Si to increase its surface area.^51^
(5) Finally, to optimize the SE material mechanical properties to reduce the stress experienced in Si and SE (at 1C, 100% SOC), several hypothetical SEs cases were simulated, and the following material design choices were proposed:
  a. If reducing maximum Si stress is of primary concern, then a moderate Young’s modulus similar to LiPON (∼77 GPa) with a low yield strength comparable to sulfide materials such as LPSCl (∼0.67 GPa) should be selected.
  b. However, if a reduction in SE stress is of greater importance, then a low Young’s modulus similar to LPSCl (∼29 GPa) with a moderate to high yield strength (1.3–3 GPa) should be adopted.
(6) Post-yield mechanical properties of different SEs should be experimentally reported as these will greatly influence the stress–strain response of the cell. A perfectly plastic post-yield behavior for the SEs was assumed in this study due to a lack of experimental values in the literature.

In summary, there has been a relative lack of emphasis on high-capacity Si NEs for SSBs and the results in this work showcase a variety of factors such as C-rate, applied pressure, and Si and SE mechanical properties that can affect the cell capacity and stress and strain evolution. The SE Young’s modulus, yield strength, and fracture properties likely play a role in the SSB cycle life and future work will consider this, in addition to other factors such as reducing the Li ion diffusion path in Si. This work set out to try to understand the mechanical cell response and the influence of SE material properties. By tailoring the SE material, it has shown the importance of SE material selection, design, and characterization of the elastic–plastic behavior, which are relevant for large format SSB systems. Electro–chemo–mechanical interactions need to be carefully considered and controlled to enable high-performance next-generation SSBs.

## Supporting Information Available

Figures showing simulations at different Li diffusion in Si values and a stress map at extremely high applied pressures to show cell capacity dependency on applied pressure (PDF)
