# Supplementary material for: Silicon-Based Solid-State Batteries: Electrochemistry and Mechanics to Guide Design and Operation
Source: ACS Appl Mater Interfaces. 2023 Aug 30;15(36):42470–80. doi: 10.1021/acsami.3c06615 (PMC10510101; doi:10.1021/acsami.3c06615)
Supplement: Supplementary file 1 — am3c06615_si_001.pdf [file am3c06615_si_001.pdf]

Supporting Information for:

Silicon-based Solid-State Batteries: Electrochemistry and Mechanics to Guide Design and Operation

*Pooja Vadhva[1], Adam M. Boyce[1,2], Anisha Patel[3], Paul R. Shearing[1,4], Gregory Offer[3,4] Alexander J. E. Rettie[1, *]*

Affiliations: 1. Electrochemical Innovation Lab, Department of Chemical Engineering, University College London, London WC1E 7JE, UK

2. School of Mechanical and Materials Engineering, University College Dublin, Dublin, D04 V1W8, Ireland

3. Department of Mechanical Engineering, Imperial College London, London SW7 1AY, UK

4. The Faraday Institution, Quad One Becquerel Avenue Harwell, Didcot OX11 0RA, UK

[*]Corresponding Author E-mail Address: a.rettie@ucl.ac.uk

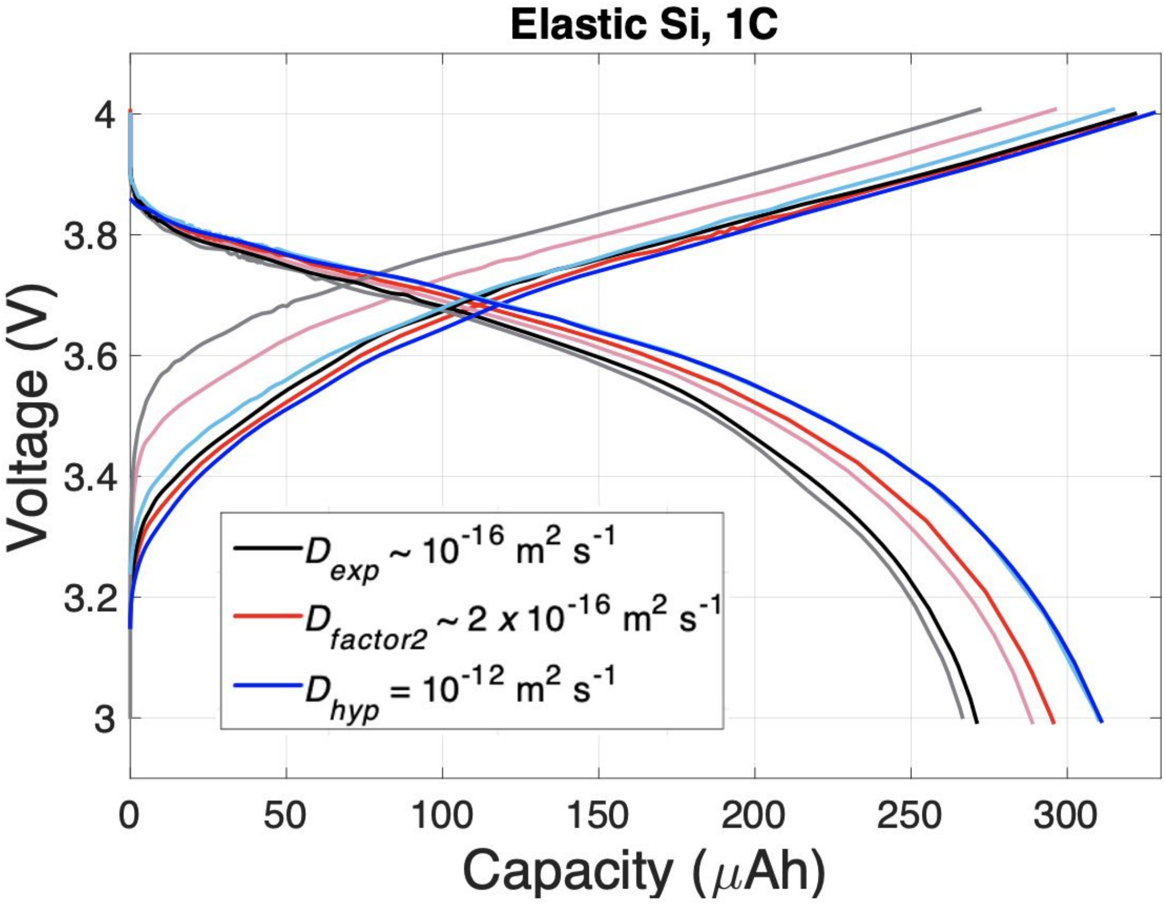

Figure S1: The discrepancy in capacity retention between cycle 1 (darker shade) and cycle 2 (lighter shade) using the experimentally extracted diffusion coefficient, D_{exp} (black line), when the value was increased by a factor of 2 ($D_{factor2}$, red line) and increased by ~4 orders of magnitude (D_{hyp}, blue line).

As the Li diffusion in Si was progressively increased from a factor of 2 to ~4 orders of magnitude, there was a progressive increase in the capacity retention and only at ~4 orders of magnitude with a hypothetical diffusion coefficient of D_{hyp} = 10^{-12} m^2 s^{-1}, was the capacity difference between cycles minimal (clearly contrasted in Figure S1).

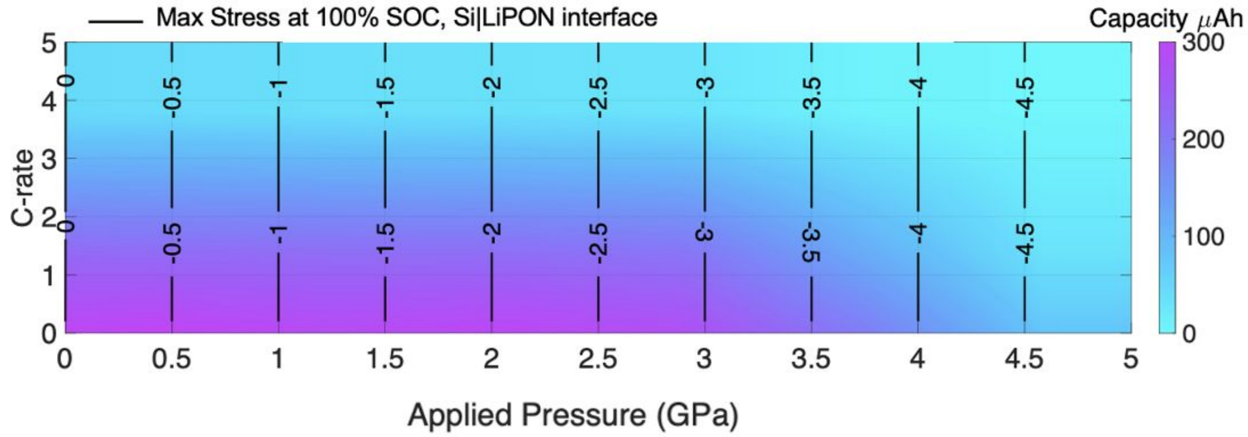

Figure S2: The dependency of averaged cell capacity on applied pressure occurs at extremely high pressures >3 GPa simulated for 100% SOC at the Si|LiPON interface.
